# Awareness and Mental Health of Male Drug Addicts With Tuberculosis During the COVID-19 Pandemic

**DOI:** 10.3389/fpsyt.2021.697508

**Published:** 2021-08-18

**Authors:** Dongming Jia, Hai Li, Yuming Xu

**Affiliations:** ^1^The Affiliated Hospital of Hangzhou Normal University, Hangzhou, China; ^2^Zhejiang Police Vocational College, Hangzhou, China; ^3^Qiongshan Mandatory Detoxification and Rehabilitation Center, Haikou, China

**Keywords:** awareness, tuberculosis, mental health, COVID-19, immunomodulation, drug addicts, rehabilitation

## Abstract

**Introduction:** At present, the COVID-19 pandemic remains the most pressing global health issue. Given the significant amount of public awareness, the infection rate and rehabilitation efforts are governed not only by the compliance of transmission mitigation strategies but also by the understanding of coexisting diseases and COVID-19 in patients with chronic infectious diseases. The main goal of this study was to evaluate the differences in demographics, as well as awareness, risk perception, and emotional reactions, among imprisoned drug addicts with and without tuberculosis (TB) regarding their perceptions of and feelings toward the COVID-19 pandemic. The secondary goal of the study was also to measure how the psychological health and nutritional indices of the drug addicts with TB changed during their ongoing rehabilitation.

**Methods:** A total of 265 male drug addicts, 45 of which were positive for TB and another 220 who were negative, were selected as subjects from a mandatory detoxification and rehabilitation center (MDRC). Data were collected through a combination of questionnaires (questions regarding COVID-19 awareness, emotional knowledge and responses, and SCL-90 tests), anthropometric examination, and laboratory blood tests, with which inferential and descriptive statistical analyses were performed.

**Results:** During a period of 1 week in early 2021, the differences in the accuracy of the responses from the questions probing the awareness of COVID-19 symptoms, transmission, susceptible populations, what kind of mask should be worn, and preventive measures between TB addicts to non-TB addicts were 11.11 vs. 60.45%, 57.78 vs. 77.27%, 66.67 vs. 78.64%, 97.98 vs. 97.73%, and 93.33 vs. 65.91%, respectively. In the risk perception and emotional reaction sections of the questionnaire, there was a significant difference in the responses to “What you were more worried about was?” (*p* < 0.001) and “Alteration in your mood since the outbreak?” (*p* = 0.002) between the two cohorts. In the section assessing the 10 dimensions of the SCL-90 scale, there were significant differences between the TB addicts and the Chinese norm. In addition, the BMI (21.06 ± 2.65 kg/m^2^) and total serum protein (77.14 ± 6.12 g/L) levels of the TB addicts were normal, but the serum hemoglobin (117.02 ± 4.97 g/L) and albumin (42.08 ± 1.81 g/L) levels were significantly lower in the TB addicts compared to the norm (*p* < 0.001).

**Conclusion:** The COVID-19 pandemic we are facing is both an epidemiologic and a psychological crisis. However, while the COVID-19 epidemic will eventually disappear (or become manageable, similar to the flu), the TB epidemic may still persist. To avoid the deleterious consequences of multiple simultaneous epidemics, complementary response measures to COVID-19 and TB can help curb the exacerbation of both situations and, therefore, save lives. Imprisoned drug addicts, especially those with TB, can master relevant knowledge on COVID-19.

## Introduction

Coronavirus disease 2019 (COVID-19), caused by the novel SARS-CoV-2 virus, emerged at the end of 2019 and caused a global pandemic. The disease mainly affects the respiratory system; however, there is growing evidence that COVID-19 is a multisystem inflammatory disease that also affects the immune system, among others (1). The pathogenesis of COVID-19 commences with a delayed antiviral response followed by an immunological overreaction that results in an excessive and prolonged proinflammatory response (2). All populations are generally susceptible to contracting COVID-19 regardless of age or sex (3), and many individuals with COVID-19 develop signs and symptoms, such as mild respiratory illness and persistent fever, an average of 5–6 days after infection with an average period of 1–14 days for symptom onset (4). After the cessation of symptoms, many COVID-19 survivors who required critical care developed psychological, physical, and cognitive impairments (5).

In 2020, the COVID-19 pandemic superseded tuberculosis (TB) as the world's leading cause of death derived from infectious diseases (6). Most TB infections are asymptomatic and latent, but approximately 5–10% of infected people with TB will develop the disease each year, with pulmonary TB (PTB) being diagnosed in the majority of these cases (7). PTB is the most common type of TB; due to the structural changes in the lungs and the suppression of cell-mediated immunity caused by long-term medication, PBT patients are prone to developing secondary lung infections, which exacerbates the PTB infection and can endanger the patient's life (8). The symptoms of PTB are similar to those of COVID-19, and patients suffering from PTB who are in close contact with COVID-19 patients have a risk of being infected with the SARS-CoV-2 virus and an even greater risk of developing serious respiratory complications from COVID-19 (9). In addition, the immune resistance of TB patients is lower compared to healthy people, and the main clinical symptoms of TB are fever, cough, and myalgia or fatigue (10), which can easily cause patients to believe they are suffering from COVID-19, thereby causing worry and fear. An observational study in Wuhan found that COVID-19 was a more-common comorbidity (36%) in patients infected with mycobacterium TB (M. TB) than diabetes (25%), hypertension (22%), ischemic heart disease (8%), and COPD (6%). They also found that M. TB coinfection was linked to more severe cases and a faster progression of COVID-19, raising concerns that latent TB infections (LTBI) represent a serious, independent risk factor that can increase the susceptibility of contracting the SARS-CoV-2 virus (11).

During the current global outbreak of COVID-19, PTB patients with compromised immune systems have a greater risk of developing complications from COVID-19, in addition to the increased exposure for hospitalized PTB patients. For the incarcerated population, the risk of infection and the incidence of TB was higher than for the general population—imprisonment and residential overcrowding being the determinants of an increased risk of latent tuberculosis infection (LTBI) among TB patients (12). In particular, more attention should be paid to older contacts to avoid infection and disease (13). There exists a higher potential for rapid TB transmission in situations where inmates actively infected with TB live in poorly ventilated custodial settings (typically in developing countries) (14). Malnutrition is another common health hazard in prisons that increases the risk of TB infection (15). In general, the link between TB and COVID-19 is likely to be bidirectional, meaning that temporary immunosuppression caused by TB infections may increase the susceptibility of patients to contracting COVID-19, and COVID-19 may in turn increase the susceptibility to TB infections (9). Among many vulnerable populations, modern imprisonment centers have a significant influence on the spread and clinical outcome of infectious diseases, making these a type of social institution that ultimately determines its own health status and outcome (16).

The state of lockdown in many parts of the world has negatively affected the physical, psychological, and emotional health of individuals (17). Despite strict measures taken by governments and communities, awareness of COVID-19 remains the most important factor in limiting its widespread transmission (18). Therefore, the best treatment against COVID-19 is by preventing it. The overall aim of this study was to investigate whether drug addicts with TB demonstrate better awareness and risk perception, as well as emotional and psychological health, related to COVID-19.

## Materials and Methods

### Sample Size

The minimum sample size needed to conduct a statistically accurate questionnaire for this investigation was 99, assuming a 10% margin of error and a 95% confidence level. Assuming a 10% attrition rate, 257 participants (50 TB addicts and 207 non-TB addicts) needed to be recruited for an 85% statistical power (α = 0.05). Meanwhile, to observe a statistically significant effect from the 45 TB addicts compared to the 220 non-TB addicts, the sample size was estimated based on an effect size (*Cohen's d*) of 0.5. Therefore, a slightly larger sample size of 265 (45 TB addicts and 220 non-TB addicts) was collected in this research study after eliminating any invalid questionnaires due to incorrect filling. This sample size was found to be sufficient based on the PASS V.15 sample size calculation software (NCSS, USA).

### Participants

We carried out a cross-sectional observational study of drug addicts in a mandatory detoxification and rehabilitation center (MDRC) in the Hainan Province of China between February 25 and March 1 of 2021 during the COVID-19 pandemic. In accordance with the Anti-Drug Law of the People's Republic of China, newly found illicit drug users of Chinese ethnicity aged 18 to 65 years, who were diagnosed by DSM/ICD with illegal drug abuse/dependence, were sent to the hospital to assess the severity of addiction, after which they received detoxification treatment under the supervision of community social workers outside of the hospital. If the drug users relapsed, they were sent to the MDRC, where they participated in a combination of detoxification treatment, physical exercise, and manual labor for 2 years (19). The drug addicts were screened for active PTB, isolated for standardized treatment, and monitored and supervised throughout the period of rehabilitation treatment in the MDRC. LTBI was diagnosed through a tuberculin skin test (TST), and active PTB was diagnosed based on chest radiography images. Participants who either refused to accept the TST test or had contraindications against the TST test, who suffered from serious diseases that affected mobility, or who were taking any type of psychotropic medications for treating mental health issues were excluded from the study.

### Protocol

The questionnaire was designed and developed based on literature precedent, expert opinion, and informal interviews with 18 drug addicts. We assured participants that the study was voluntary, anonymous, and confidential. Included in the questionnaire was a statement that the purpose of the study was to identify the participants' feelings or experiences related to the topic of investigation. In addition, some questions within necessitated only binary answers (yes or no), and there were no “neutral/don't know” answers. The questionnaire included three parts. The first part consisted of demographic information, such as age, marital status, education, employment status, as well as smoking, diet, and physical activity habits. The second part asked about the participants' level of awareness (Q1–Q5), as well as risk perception and emotional reaction (Q1–Q3), in accordance with the clinical guideline “Diagnosis and Treatment Protocol for Novel Coronavirus Pneumonia” (Trial Fifth Edition, 2020, China). The possible answers to these questions are shown in **Table 2**. In the third part, we used the Symptom Check List 90 (SCL-90, revised Chinese version) introduced in 1984 to assess the psychological health status of the participants. This scale produced 10 primary symptom dimensions: somatization (SOM), anxiety (ANX), obsessive-compulsive disorder (OCD), depression (DEP), interpersonal sensitivity (IS), psychoticism (PSY), paranoid ideation (PAR), hostility (HOS), phobic anxiety (PHOB), and general severity index (GSI). The participants were required to report their psychological health status in the week prior to the questionnaire. After completing the series of questions and obtaining written consent, blood samples (20 mL) were collected from each participant in the study, which were used to measure hemoglobin, total serum protein, and albumin levels as nutritional indices. In addition, the BMIs (body mass indices) of the participants were recorded.

### Statistical Analyses

We hypothesized that awareness and mental health differences existed between the TB and non-TB groups. To test this hypothesis, inferential and descriptive statistical analyses were performed based on the data gathered from the drug addict participants in the study, including COVID-19 awareness, risk perception, and emotional reactions, as well as psychological health status, anthropometric measures, and results from the blood tests. All of the data were collected and organized using Excel 2016 (Microsoft Corporation, Redmond, WA, USA) and transferred to SPSS version 25.0 (IBM SPSS Statistics for Windows, IBM, Armonk, NY, USA, 2017) for statistical analysis. Descriptive statistics were used for all variables. The data reported herein were presented as the mean ± SD as either a rate or constituent ratio (%). Comparisons of the data corresponding to the demographics, level of awareness, risk perception, and emotional reactions of the drug addicts were performed using both the χ^2^ and Fisher's exact tests. In contrast, comparisons of the SCL-90 dimensions, BMI, and blood test data between the TB addicts and the non-TB addicts were performed using Welch's *t*-test. In the bilateral test, the statistical significance was indicated as *p* < 0.05.

## Results

The demographics of the drug addicts in this study are shown in Table 1. The participants varied in age from 18 to 65 years (mean 36.27 ± 13.1 years), and 31.32% of the 265 participants were 35 years old and younger. There were no significant differences between the TB and non-TB addicts with respect to age (*p* = 0.287), marital status (*p* = 0.267), and employment status (*p* = 0.060); however, a significant difference in the education (*p* = 0.002) of the TB and non-TB addicts was observed. Compared to the non-TB addicts, the TB addicts had unhealthy lifestyle habits, such as a high-salt diet (*p* < 0.001) and lack of physical activity (*p* < 0.001), and almost all addicts (99.62%) smoked.

**Table 1:** Demographics of the study participants.

**Characteristics**	**Total (265)**	**TB (45)**	**Non-TB (220)**	*****χ*****^2^****	***P***
	***N* (%)**	***N* (%)**	***N* (%)**		
Age (years)				2.500	0.287
18–34	83 (31.32)	11 (24.44)	72 (32.73)		
35–54	177 (66.79)	32 (71.11)	145 (65.91)		
≥55	5 (1.89)	2 (4.44)	3 (1.36)		
Marital status				2.638	0.267
Married	104 (39.25)	13 (28.89)	91 (41.36)		
Divorced	130 (49.06)	25 (55.56)	105 (47.73)		
Single	31 (11.70)	7 (15.56)	24 (10.91)		
Education				14.761	0.002
Primary school	117 (44.15)	30 (66.67)	87 (39.55)		
Junior high school	117 (44.15)	10 (22.22)	107 (48.64)		
Senior high school	18 (6.79)	3 (6.67)	15 (6.82)		
University and above	4 (1.51)	2 (4.44)	2 (0.91)		
Employment status				7.076	0.060
Unemployed	186 (70.19)	39 (86.67)	147 (66.82)		
Self-employed	25 (9.43)	1 (2.22)	24 (10.91)		
Entertainment industry	9 (3.40)	1 (2.22)	8 (3.64)		
Semi-employed	45 (16.98)	4(8.89)	41 (18.64)		
Smoking				0.092	0.762
Yes	264 (99.62)	44 (100.00)	219 (99.55)		
No	1 (0.38)	1 (0.00)	1 (0.45)		
High-salt diet				13.121	<0.001
Yes	198 (74.72)	24 (53.33)	174 (79.09)		
No	67 (25.28)	21 (46.67)	46 (20.91)		
Physical activity				21.270	<0.001
Inactive to light	221 (83.40)	26 (57.78)	195 (88.64)		
Moderate	40 (15.09)	17 (37.78)*^*b*^*	23 (10.45)		
Vigorous	4 (1.51)	2 (4.44)	2 (0.91)		

Table 2 presents the results of the questions regarding the awareness of the COVID-19 pandemic. The awareness accuracy rates of Q1–Q5 in the survey for the TB addicts and non-TB addicts were 11.11 vs. 60.45%, 57.78 vs. 77.27%, 66.67 vs. 78.64%, 97.98 vs. 97.73%, and 93.33 vs. 65.91%, respectively. There were no significant differences in the responses to Q3 (susceptible population) (*p* = 0.168) between the TB and non-TB addicts, and more than 97% knew that a surgical mask should be worn and would be effective at preventing transmission of COVID-19 (Q4). In addition, the non-TB addict group had adequate knowledge of Q1 (typical symptoms) (*p* < 0.001), Q2 (transmission routes) (*p* = 0.003), and Q5 (protective measures) (*p* = 0.002).

**Table 2:** COVID-19 awareness results.

**Survey question (single-choice items)**	**TB (45)**	**Non-TB (220)**	*****χ*****^2^****	***P***
	***N* (%)**	***N* (%)**		
Q1. Symptoms of COVID-19?			48.261	<0.001
Fever	32 (71.11)	48 (21.82)		
Cough	4 (8.89)	15 (68.19)		
Myalgia or fatigue	4 (8.89)	24 (10.91)		
All above	5 (11.11)	133 (60.45)		
Q2. Transmission routes of COVID-19?			12.312	0.003
Contact	12 (26.67)*^*a*^*	16 (72.73)		
Droplet	7 (15.56)*^*a, b*^*	34 (15.45)		
Both	26 (57.78)*^*b*^*	170 (77.27)		
Q3. Susceptible population?			3.656	0.168
All people	30 (66.67)	173 (78.64)		
Only the elderly and children	5 (11.11)	22 (10.00)		
Except for young people	10 (22.22)	25 (11.36)		
Q4. What kind of mask should be worn?			0.000	1.000
General cotton mask	1 (2.22)	5 (2.27)		
Medical surgical mask	44 (97.78)	215 (97.73)		
Q5. Which protective measures should you choose?			17.262	0.002
Drink plenty of Radix Isatidis	1 (2.22)	25 (11.36)		
Steaming vinegar	1 (2.22)	9 (4.09)		
Wearing multi-layer mask	1 (2.22)	18 (8.18)		
Wash hands frequently, go out less, and do not gather	42 (93.33)	145 (65.91)		

The results of the risk perception and emotional reaction questions are shown in Table 3. The survey questions in this section included multiple choices. In response to Q1 (What you were more worried about was?), significantly more non-TB addicts (55.00%) chose the answer “People around me are NOT well protected” compared to the TB addicts (*p* < 0.001), but the TB addicts were more worried about “New and confirmed local cases” (66.57%). In response to Q3 (Alteration in your mood since the outbreak?), the choice of “Nervous at first, but now I'm relieved” and “Worried about being infected because of health” had significant differences (*p* = 0.002). However, there were no significant differences between the two cohorts in the responses to Q2 (*p* = 0.114).

**Table 3:** Risk perception and emotional reaction results.

**Survey question (multiple-choice items)**	**TB**	**Non-TB**	*****χ*****^2^****	***P***
	***N* (%)**	***N* (%)**		
Q1. What you were more worried about was?			18.833	<0.001
New and confirmed local cases	30 (66.57)	52 (23.64)		
Number of deaths	7 (15.56)	48 (21.82)		
Shortage of personal protective equipment	22 (48.89)	56 (25.45)		
People around me are NOT well protected	21 (56.67)	121 (55.00)		
I will be infected with COVID-19	26 (57.78)	94 (42.73)		
Q2. Your judgment on the current epidemic situation?			5.959	0.114
Government has strong control	40 (88.89)	167 (75.91)		
Try to get vaccinated	32 (71.11)	193 (87.73)		
Proper protective measures for the public	41 (91.11)	132 (60.00)		
Life is gradually back on track	38 (84.44)	152 (69.09)		
Q3. Alteration in your mood since the outbreak?			14.955	0.002
Nervous at first, but now I'm relieved	36 (80.00)	187 (85.00)		
Have been nervous, afraid of the rebound	2 (4.44)	30 (13.64)		
Worried about being infected because of health	19 (42.22)	34 (15.45)		
Always believe that if you do a good job of protection, you will not get infected	40 (88.89)	189 (85.91)		

The comparisons of the SCL-90 data between TB addicts and Chinese norm are shown in Table 4. The *t*-test results showed that the scores of the 10 dimensions of the SCL-90 scale in the TB addict group (*n* = 45) were more significant than those of the Chinese norm (*n* = 12,160) (20), indicating that the mental health status of the TB addicts was clearly worse than the norm. Among them, the ANX (*t* = 9.273) and DEP (*t* = 8.295) dimensions had the largest deviations between the TB addicts and the Chinese norm.

**Table 4:** SCL-90 scores (mean ± SD).

**Dimension**	**Norm** **(*n* = 12,160)**	**TB** **(*n* = 45)**	***t***	***Cohen's d***	***p***
SOM	1.37 ± 0.46	1.83 ± 0.51	−6.693	−1.000	<0.001
ANX	1.51 ± 0.55	2.12 ± 0.44	−9.273	−1.110	<0.001
OC	1.66 ± 0.58	1.77 ± 0.30	−2.443	−0.190	0.019
DEP	1.45 ± 0.53	1.86 ± 0.33	−8.295	−0.774	<0.001
IS	1.51 ± 0.55	1.73 ± 0.32	−4.587	−0.401	<0.001
PSY	1.34 ± 0.44	1.81 ± 0.51	−6.174	−1.068	<0.001
PAR	1.41 ± 0.50	1.49 ± 0.25	−2.131	−0.160	0.039
HOS	1.48 ± 0.57	1.68 ± 0.26	−5.115	−0.351	<0.001
PHOB	1.23 ± 0.39	1.64 ± 0.42	−6.538	−1.051	<0.001
GSI	1.51 ± 0.58	1.83 ± 0.24	−8.849	−0.553	<0.001

The nutritional indices results are presented in Table 5. There was no significant difference in the BMI (*p* = 0.633) and total serum protein levels (*p* = 0.487) between the TB addicts and the Chinese norm, but the hemoglobin and albumin levels were significantly lower in the TB addict group compared to the norm (*p* < 0.001). The effect sizes related to the differences were generally large (*Cohen's d* > 0.3).

**Table 5:** Nutritional indices (mean ± SD).

**Nutritional indices**	**Norm (ranges)**	**TB** **(*n* = 45)**	***t***	***Cohen's d***	***p***
BMI (kg/m^2^)	18.5–24.0	21.06 ± 2.65	0.481	0.072	0.633
Hemoglobin (g/L)	120.0–160.0	117.02 ± 4.97	31.017	4.618	<0.001
Total protein (g/L)	66.0–87.0	77.14 ± 6.12	−0.702	−0.100	0.487
Albumin (g/L)	38.0–51.0	42.08 ± 1.81	8.969	1.341	<0.001

## Discussion

The aim of this study was to explore the feelings and experiences of drug addicts with TB toward the COVID-19 pandemic, which were evaluated by assessing the responses of the drug addicts to questions regarding various physical, psychological, and emotional health parameters, such as awareness of COVID-19, risk perception, emotional reactions, and SLC-90 scores, as well as the blood serum levels of certain proteins and BMIs (nutritional status). We found that awareness and mental health of the drug addicts with TB were significantly influenced by COVID-19. Among the 265 drug addicts who participated in this study, the number of non-TB addicts (*n* = 220) who answered the questions regarding the symptoms of COVID-19 correctly was much higher than the number of TB addicts (*n* = 45). Most TB addicts chose fever symptoms, but this might have been influenced by their knowledge of the symptoms of fever due to TB. The accuracy of the answers to the question regarding the transmission of COVID-19 (i.e., transmitted through surface contact and fluid droplets) was very high in both groups. In addition, more than 97% of the participants correctly answered “medical surgical mask” to the question regarding “What kind of mask should be worn?” to prevent COVID-19 transmission. Among the “protective measures” question, the number of non-TB addicts who correctly responded with “wash hands frequently, go out less, and do not gather” was significantly lower than the corresponding accuracy of the TB addicts group; however, some non-TB addicts chose to exercise and get a decent amount of sleep, which was consistent with the options in the physical activity questions in the demographics section of the questionnaire (21). The total amount of physical activity of the non-TB addicts was significantly higher than that of the TB addicts, which was justifiable because physical activity typically declines as a result of TB infection due to the impairment of pulmonary function (22).

With regard to risk perception and emotional reactions, the TB addicts were more worried about “new and confirmed local cases” and “I will be infected with COVID-19,” while non-TB addicts were more worried about “people around me are NOT well protected.” This might have been attributed to TB drug addicts being more concerned about their poor physical condition compared to non-TB addicts (22). When asked about their judgment on the current epidemic situation, the non-TB drug addicts were more likely to pin their hopes on “getting vaccinated,” but TB addicts more consistently chose the “proper protective measures for the public” response. However, both groups generally believed that the government has a strong control of the current situation. When caring for TB patients, the primary task is to provide anti-tuberculosis treatment, after which TB patients can be vaccinated when they recover or are cured (23). When asked about how the moods of the participants were altered since the coronavirus outbreak, most of the answers for all of the participants were either “nervous at first, but now I'm relieved” and “always believe that if you do a good job of protection, you will not get infected.” This indicated that the participants had overall positive attitudes about current control measures in China.

Historically, pandemics have elicited a tremendous amount of fear. Ordinary citizens may experience some psychological changes, such as fear, depression, anxiety, psychological turbulence, or uncertainty (24). Comparably, drug abusers often endure excessive stress during compulsory detoxification treatment, which is the major drug rehabilitation modality in China currently, leading to a high prevalence of anxiety symptoms (25). A previous study in China showed that the SCL-90 scale (Chinese version) was used to evaluate the psychological health of eligible drug addicts to compare with normal adults, and the results showed that the psychopathological conditions of comorbidities based on the SCL-90 scale were more severe in all of the 10 dimensions assessed in the drug addicts compared to the normal adults (26). In our study, we also came to similar conclusions, with anxiety and depression being the two dimensions with the highest scores of the 10 that were assessed. China uses the media and the internet to disseminate information related to the epidemic to the public in a timely manner to help alleviate the negative reaction of the public.

The impact of SARS-CoV-2 on long-term diseases, such as TB, is of particular concern because TB is still a serious public health concern throughout the world, especially in emerging economies (23). In 2019, TB was the leading cause of death from a single infectious pathogen worldwide. In China, the number of people who developed TB in 2019 accounted for 8.4% of the global total. In some countries, TB was the leading cause of death in prisons, mainly due to poor physical conditions, overcrowding, and lack of proper treatment (22). The risk of TB exposure and transmission in the prison population is more serious than in other environments; therefore, there are many challenges that preclude its prevention and treatment. There is a variety of complex and unstable factors that govern the transmission of TB between drug users and ordinary social groups because of the two-way flow between these two populations, such that the normal population can enter detention centers, and drug addicts can also return to society. Meanwhile, staff, visitors, and drug abusers in detention centers also may come into direct contact with drug addicts with TB, resulting in increased risk of transmission (27).

In addition to other risks, malnutrition is closely related to the incidence of TB. Protein-calorie malnutrition (PCM) is a risk factor of TB and affects TB treatment outcome (16). Low BMIs are associated with a large percentage of people with TB in developing countries. One study found that the risk of developing TB in the low weight group (BMI <18.5) was 12.4 times higher than in the normal weight group (18.5 ≤ BMI <25.0) (28). In our study, the BMI and total protein levels of TB addicts were normal, while the levels of hemoglobin and albumin in the blood were lower than average for normal Chinese people. We speculated that a reasonable diet and different diet plans according to the clinical symptoms of drug abusers can effectively improve the BMI and total protein levels. Low serum albumin levels are typically a consequence of poor nutrition, but they can also be related to abnormal liver function, as plasma proteins are biosynthesized in the liver. In addition, excessive high-calorie and high-protein diets also increase the burden on the liver. Anemia is also a condition that plagues TB patients, the pathogenesis of which is related to the inhibition of erythropoiesis through nutritional deficiency, the expression of inflammation markers, and malabsorption syndromes (29). Given these perspectives, if the world expects to eliminate the public health threat of TB by 2035, it must address the main drivers of tuberculosis: malnutrition, diabetes, poverty, smoking, and household air pollution (6).

## Limitations

Our study had several limitations that should be emphasized. TSTs are widely used for TB screening, but increased age, the use of immunosuppressive therapies, and high BMI were also associated with producing false negative TB diagnoses (30). This study paid more attention to the awareness, risk perception, and emotional reactions that drug addicts with TB may have regarding COVID-19 because any abnormalities in the dimensions assessed in the SCL-90 test may be associated with psychological phenomena that are common among drug abusers. However, whether the COVID-19 pandemic had adverse psychological effects on large populations was not studied in this paper. We will further increase the number of participants, including female drug addicts, to understand the psychological development of this cohort, and we will conduct in-depth research on the nutritional health and the consistency of the nutritional cognition and behavior of the TB addicts, which can be used to more-effectively promote the rehabilitation of TB addicts and prevent the omnipresent spread of TB.

## Conclusion

This study supported the claim that the ongoing COVID-19 pandemic is both an epidemiologic and psychological crisis. The psychologic and social consequences caused by the virus may be as equally destructive as the infection itself (22). Imprisoned drug addicts, especially those with TB, can master relevant knowledge on COVID-19. The risk perception and emotional reactions among TB addicts toward COVID-19 have begun to gradually return to normal since the pandemic outbreak began in early 2020. Most TB addicts have poor mental health, with anxiety and depression being the most common mental health issues in these patients. Therefore, routine medical follow-ups, including physical and psychological evaluations and guidance, are significantly beneficial to improve patients' awareness and prevent not only psychological crises but also TB transmission.

The susceptible population with PTB consists of addicts with low immune function. In addition to routine drug therapy, detention centers (i.e., institutions, such as prisons and MDRCs) in China also perform nutritional management for TB addicts. Quarantining TB addicts in these institutions, to some extent, is a public health measure that can reduce the risk of infection and death from TB. To corroborate this measure, no healthcare staff have since been infected with COVID-19 in imprisonment centers, and no TB patients have been co-infected with COVID-19.

The Chinese government, the National Health Commission, and relevant departments must promptly and transparently announce any treatment progress, protective measures, and future outlooks regarding the COVID-19 pandemic to the public. To avoid another global pandemic, the current COVID-19 pandemic must disappear; however, the global TB epidemic will still exist, at least for the time being. If we utilize the same response measures for mitigating the spread of TB as we do for COVID-19, we can help curb the exacerbation of both diseases and, therefore, save lives. Although the COVID-19 pandemic has changed the way people live and behave, the measures that the global society implemented to fight it will provide useful information for managing the spread of TB infections and the exacerbation of TB epidemics in the future.

## Data Availability Statement

The raw data used for statistical analysis in this study will be made available by the authors without undue reservation.

## Ethics Statement

The studies involving human participants were reviewed and approved by the ethical commission of the Hainan Provincial Administration of Detoxification. The investigation was in accordance with the latest version of the Declaration of Helsinki. The participants provided their written informed consent to participate in this study.

## Author Contributions

DJ and HL designed the study, DJ conducted the statistical analyses and took the lead in writing the manuscript, and YX contributed significantly to the interpretation of the results. All authors contributed to the article and approved the submitted version.

## Conflict of Interest

The authors declare that the research was conducted in the absence of any commercial or financial relationships that could be construed as a potential conflict of interest.

## Publisher's Note

All claims expressed in this article are solely those of the authors and do not necessarily represent those of their affiliated organizations, or those of the publisher, the editors and the reviewers. Any product that may be evaluated in this article, or claim that may be made by its manufacturer, is not guaranteed or endorsed by the publisher.
